# Pericardial effusion – an unusual manifestation of salmonellosis: a case report

**DOI:** 10.1186/1757-1626-1-375

**Published:** 2008-12-06

**Authors:** Manisa Sahu, Sistla Sujatha, Chaya DR, Subhash Chandra Parija

**Affiliations:** 1 Department of Microbiology, Jawaharlal Institute of Postgraduate Medical Education and Research, Puducherry, India

## Abstract

**Background:**

Nontyphoidal salmonellae are important food-borne pathogens that are usually associated with self-limiting gastroenteritis. Occurrence of extra-intestinal non-typhoidal salmonellosis in humans is increasing in many developing countries. The risk of extra-intestinal nontyphoidal salmonellosis is higher in patients with impaired cell mediated immunity, lympho-proliferative disorders and IL-12 deficiencies. Pericardial involvement is one of the rare manifestations accounting for <2% cases but the mortality is very high.

**Case presentation:**

A 23 year old male was admitted in the medicine ward with complaints of fever, chest pain with non-productive cough, not associated with hemoptysis for past 3 weeks. He was a known case of Hodgkin's lymphoma and was treated with complete course of chemotherapy. Cardiovascular examination, chest X-ray and ECG findings suggested this to be a case of pericardial effusion. The causative agent of this purulent pericardial effusion was identified as Group B Salmonella following isolation from the pericardial fluid.

## Background

Salmonellae are a group of Gram-negative bacilli that can cause a wide spectrum of diseases both in animals as well as humans. *Salmonella *Typhi and *Salmonella *Paratyphi A are restricted to human hosts, in whom these organisms cause enteric (typhoid) fever. The other serotypes referred to as non-typhoidal salmonellae (NTS), can colonize the gastrointestinal tracts of a broad range of animals and accidentally infect human beings. Non-typhoidal salmonellae are important food-borne pathogens associated with large out breaks of salmonellosis occurring in situations of poor hygiene and improper food processing [1,2]. Cases of extra-intestinal NTS are being increasingly reported in literature particularly in patients with impaired cell mediated immunity, lympho-proliferative disorders and IL-12 deficiencies [3]. Pericardial involvement though rare has a very high mortality rate [4]. Here we report a case of pericarditis in a 23 yr old male with Hodgkin's lymphoma due to Group B Salmonella.

## Case presentation

A 23 year old Muslim male was admitted in the medicine ward in August 2007 with complaints of fever, chest pain with non-productive cough, not associated with hemoptysis for past 3 weeks. He was a known case of Hodgkin's lymphoma and was treated with complete course of chemotherapy. On examination, patient was febrile, had tachycardia and tachypnoea. Jugular venous pressure was raised. Supraclavicular lymph nodes were enlarged matted and non tender. There was no peripheral oedema. Abdominal examination revealed no organomegaly. On auscultation, heart sounds were found to be diminished. Total leukocyte count was 11,300/c.mm – the differential count showed 70% neutrophils, 24% lymphocytes and 4% eosinophils. Erythrocyte sedimentation rate (ESR) was 32 mm at the end of one hour by Westergren's method. Chest roentgenogram showed cardiomegaly. In Electrocardiogram (ECG) there was widespread elevation of the ST segments and a reduction in voltage in QRS complexes. It was diagnosed as a case of pericardial effusion. Pericardial fluid & blood were sent for culture. Direct smear examination of pericardial fluid showed pus cells without any bacteria. On MacConkey agar pericardial fluid yielded non lactose fermenting colonies after overnight incubation. The organism was identified as *Salmonella *by standard biochemical tests and was confirmed as Group B Salmonella by agglutination with polyvalent O antisera (Poly O) and specific O4 antisera. The same organism was isolated from a repeat sample of pericardial fluid. Blood culture was negative. The isolate was sensitive to gentamicin, amikacin, ceftriaxone ceftazidime, ciprofloxacin and ampicillin. The patient survived after treatment with intravenous ceftriaxone (2 gm intravenously twice daily for two weeks) and other supportive therapy.

## Discussion

Pericarditis & Pericardial effusion are the most common pathologic processes involving the pericardium. Acute pericarditis is a life threatening condition. It can either be due to an infectious or a non infectious cause. Common infectious causes of pericarditis are *Streptococcus pneumoniae*, other *Streptococcus *species, and *Staphylococcus aureus *etc [5].

While salmonellosis is often considered to affect primarily the gastrointestinal tract, bacteremia and focal extra intestinal infections like meningitis, empyema, and pericarditis do occur especially in immuno-compromised patients. We reported a case of meningitis due to Group B *Salmonella*, the organism was isolated from CSF, blood as well as stool sample, the peculiarity was the stool isolate that was ESBL producing where as the CSF and blood isolates were sensitive to all the antibiotics [6].

Pericarditis is a rare complication of *Salmonella *infections, only 30 cases being reported so far [7]. The mortality was very high in these cases [4]. Transient salmonella bacteremia from the gastrointestinal tract has also been previously noted [8]. The organism finally gets restricted to the organ (pericardium) and keeps multiplying.

The first case of nontyphoidal *Salmonella *pericarditis was reported in a 36-year-old woman by Cohen et al [9]. Another case report was from a 42 yr male who had pericarditis following bacteremia after an episode of gastroenteritis [2]. *Salmonella typhimurium *is the most common organism isolated in nontyphoidal suppurative pericarditis, accounting for 50% of the reported cases [10]. Most other cases are due to *Salmonella enteritidis *[7]. Salmonella pericarditis has also been reported in SLE patients on dialysis due to defect in host defense against salmonella. Few case reports of co-existence of pericarditis with empyema are there which is very rare [9].

Our case was a known case of Hodgkin's disease treated with chemotherapy certifying the compromised immune status of the patient. Later in the course he developed pericardial effusion due to Group B Salmonella and had a favorable outcome with conservative treatment with antibiotics.

To the best of our knowledge this is one of the very few case reports of purulent pericardial effusion due to Group B Salmonella from India.

## Abbreviations

NTS: nontyphoidal salmonellae; ESR: erythrocyte sedimentation rate; ECG: electrocardiogram; CSF: cerebrospinal Fluid; ESBL: extended spectrum beta lactamases; SLE: systemic lupus erythematosus.

## Consent

Written informed consent was obtained from the patient for publication of this case report. A copy of the written consent is available for review by the Editor-in-Chief of the journal.

## Competing interests

The authors declare that they have no competing interests.

## Authors' contributions

MS conducted the literature review and participated in preparation of the manuscript. SS participated in preparation of the manuscript and review of the patients' medical records. CDR participated in the review of patients' medical records. SCP participated in preparation of the manuscript All authors read and approved the final manuscript.
